# Evaluation of the effectiveness and mechanism of action of the Chang-Kang-Fang formula combined with bifid triple viable capsules on diarrhea-predominant irritable bowel syndrome

**DOI:** 10.3389/fmicb.2023.1160783

**Published:** 2023-06-27

**Authors:** Jing Sun, Mengqiu Zhang, Wei Liu, Youqian Liu, Dongjian Zhang, Xinyu Fan, Jian Zhang, Tian Li, Min Lu

**Affiliations:** ^1^Department of Central laboratory, Nanjing Lishui District Hospital of Traditional Chinese Medicine, Nanjing, China; ^2^Department of TCMs Pharmaceuticals, School of Traditional Chinese Pharmacy, China Pharmaceutical University, Nanjing, China; ^3^School of Third Clinical Medicine, Nanjing University of Chinese Medicine, Nanjing, China; ^4^Suqian Hospital of Traditional Chinese Medicine, Suqian, China; ^5^Department of Gastroenterology, Nanjing Lishui District Hospital of Traditional Chinese Medicine, Nanjing, China; ^6^Affiliated Hospital of Integrated Traditional Chinese and Western Medicine, Nanjing University of Chinese Medicine, Nanjing, China

**Keywords:** diarrhea-predominant irritable bowel syndrome, Chang-Kang-Fang, bifid triple viable capsule, serotonin, intestinal microbiota

## Abstract

**Introduction:**

The Chang-Kang-Fang (CKF) formula, a traditional Chinese herbal formula, can decrease serotonin (5-HT) levels and treat irritable bowel syndrome (IBS). Probiotics have a better synergistic effect on diarrhea-predominant IBS (IBS-D) when combined with 5-HT_3_ receptor antagonists. The present study aimed to elucidate the efficacy and the mechanisms of action of the CKF formula combined with bifid triple viable capsules (PFK) against IBS-D.

**Methods:**

The rat models of IBS-D were induced by gavage with senna decoction plus restraint stress. The CKF formula, PFK and their combination were administered to the rats. Their effects were evaluated based on general condition of the rats and the AWR score. The levels of 5-HT and fos protein in the colon and hippocampus were measured by immunohistochemistry. The levels of SP and VIP, as well as ZO-1 and occludin in the colon, were determined by enzyme-linked immunosorbent assay and immunohistochemistry. The intestinal microbiota in faeces was analyzed by 16S rRNA high-throughput sequencing.

**Results:**

The results showed that the oral CKF formula combined with PFK (CKF + PFK) could significantly relieve the symptoms of IBS-D, including elevating the weight rate and decreasing the AWR score. Compared with the MC group, administration of CKF + PFK significantly reduced the expression of fos in the colon and hippocampus and that of 5-HT, SP and VIP in the colon and increased the levels of 5-HT in the hippocampus and ZO-1 and occludin in the colon. The above indexes exhibited statistical significance in the CKF + PFK group relative to those in the other groups. Moreover, treatment with CKF + PFK improved the diversity of intestinal microbiota and the abundance of *Firmicutes*, *Lachnospiraceae* and *Ruminococcaceae* but decreased those of *Bacteroidetes* and *Prevotellaceae*.

**Conclusions:**

The CKF formula combined with PFK may have a synergistic effect on IBS-D by slowing gastrointestinal motility, lowering visceral hypersensitivity, enhancing the intestinal barrier function and modulating the composition of intestinal microbiota.

## 1. Introduction

Irritable bowel syndrome (IBS) is a chronic functional gastrointestinal tract disorder without organic abnormalities. Currently, the prevalence of IBS is approximately 1–16% in China and 10–18% in Western countries (Choung and Locke, 2011; Lu et al., 2014). According to the Rome IV criteria (Altomare et al., 2021), diarrhea-predominant irritable bowel syndrome (IBS-D) is one of the most common clinical subtypes accounting for 40% of all IBS cases (Liang S.-B. et al., 2019). IBS-D usually manifests as abdominal pain or diarrhea accompanied by alteration of bowel habits and mental dysfunction. Patients with IBS-D commonly have an adverse mental state that significantly influences their quality of life due to persistent and relapsing episodes. This can directly or indirectly lead to a high medical burden and increase the risk of comorbidities (e.g., psychological and psychiatric diseases) (Lacy et al., 2017, 2019; Cibert-Goton et al., 2021).

Patients with IBS-D usually present with multiple issues, such as gastrointestinal (GI) motility disorder, visceral hypersensitivity, intestinal immune activation, intestinal microbiota dysbiosis and intestinal barrier dysfunction (Ishaque et al., 2018). Clinical studies have implicated that patients with IBS-D have significantly high serotonin (5-HT) levels (Yu et al., 2016). 5-HT is an essential neurotransmitter and a paracrine signaling molecule that regulates GI function and is located predominantly in the enterochromaffin (EC) cells, where it accounts for 90% of the total body levels (Moore et al., 2013). 5-HT acts on nerve endings within the submucosa and mucosa via a range of receptors (including 5-HT_3_ receptors) to stimulate motility (Marciani et al., 2010). Studies have shown that abnormalities in the 5-HT signaling system can trigger GI motility disorders and visceral hypersensitivity and eventually result in the development of IBS-D (Zhu et al., 2017).

5-HT_3_ receptor antagonists have been exploited as conventional medications for IBS-D to target the main bowel symptoms (Roberts et al., 2020). However, their use is frequently symptom-based and is associated with severe adverse effects, such as recurrent abdominal pain and constipation (Sun et al., 2015). Research has demonstrated that patients with IBS-D receiving 5-HT_3_ receptor antagonists often achieve limited improvement and experience common side effects, such as constipation and nausea, as well as ischemic colitis (Zheng et al., 2017). Therefore, a majority of patients with IBS-D increasingly turn to alternative treatments (Koloski et al., 2003; van Tilburg et al., 2008). Recently, Chinese Herbal Medicines (CHMs) have been considered safe and curative for several diseases and have been extensively applied to treat diarrhea for thousands of years (Ko et al., 2011; Kwak et al., 2014; Compare et al., 2017; Bu et al., 2020). The Chang-Kang-Fang (CKF) formula, a traditional Chinese herbal formula, has been used clinically in China for the treatment of IBS (especially IBS-D). This formula consists of seven common crude herbs (Table 1). Previous clinical and preclinical trials revealed that the CKF formula reduced intestinal motility and visceral hypersensitivity by decreasing the 5-HT levels with few side-effects (Li and Shen, 2015; Jie et al., 2020).

**Table 1:** The ingredients of Chang-Kang-Fang formula.

Chinese name	Latin binomial nomenclature	English name	Part of use	Amount (g)
Bai Shao	*Paeonia lactiflora* Pall	Paeoniae Alba Radix	root	15
Fang Feng	*Saposhnikovia divaricata* (Trucz.) Schischk	Saposhnikoviae Radix	root	6
Shu Di Huang	*Rehmannia glutinosa* (Gaertn.) Libosch	Rehmanniae Radix	root	5
Tu Si Zi	*Cuscuta chinensis* Lam	Cuscutae Semen	seed	5
Huang Lian	*Coptis chinensis* Franch	Coptidis Rhizoma	rhizome	3
Jin Qiao Mai	*Fagopyrum dibotrys* (D. Don) H. hara	Fagopyri Dibotryis Rhizoma	rhizome	8
Chan Tui	*Cryptotympana pustulata* Fabricius	Periostracum Cicadae	exuviae	3

However, in most cases, a single drug cannot completely cure IBS-D caused by multiple factors. Drug combinations can produce a synergistic effect, improve efficacy and reduce side effects. A study reported that probiotics could enhance the efficacy of ondansetron (a 5-HT_3_ receptor antagonist) in intestinal disease (Schnadower et al., 2015). Several clinical trials have shown that probiotics can be used for the prevention of IBS-D and demonstrate a better synergistic effect (Sánchez et al., 2017; Zhao, 2018; Jiang et al., 2020). Accordingly, whether probiotics can enhance the efficacy of CHMs in treating IBS-D is worthy of further investigation.

Bifid triple viable capsule (product name: Pei-Fei-Kang, PFK) is a probiotic mixture and has been shown to be effective in IBS-D. It comprises three bacterial species, including *Bifidobacterium longus, Lactobacillus acidophilus and Enterococcus faecalis* (Chen et al., 2019). The US-based International Scientific Association for Probiotics and Prebiotics (Pham et al., 2021) recommends the use of probiotics in IBS before the use of licensed drugs, considering its inexpensiveness (Liang D. et al., 2019), absence of side effects (Tarrerias et al., 2011), and safety (Preston et al., 2018).

**Figure 1:**
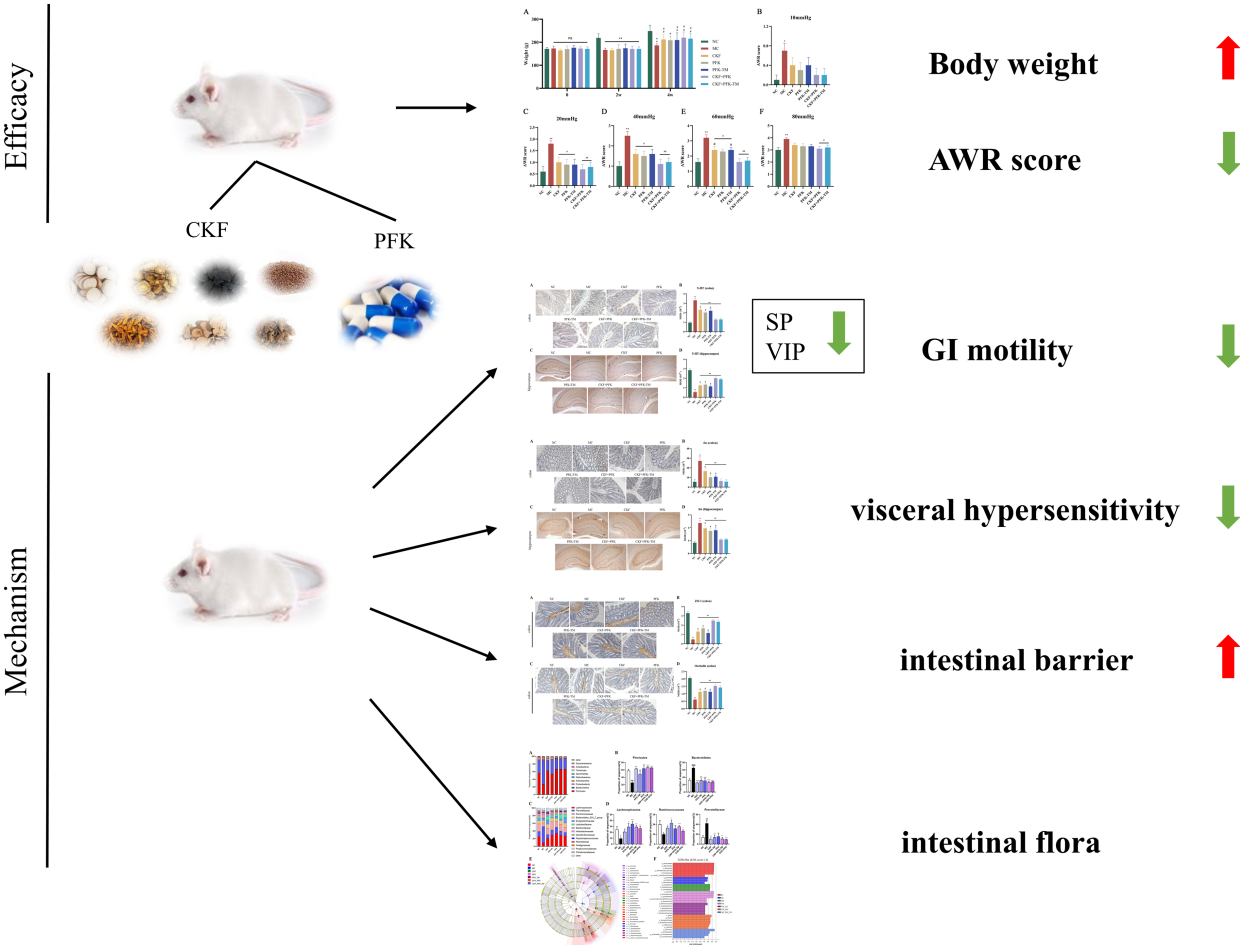
The summary of the protective effect CKF combined with PFK on IBS-D.

Hence, we speculated that the combination of the CKF formula with PFK might exert complementary therapeutic effects to ameliorate the clinical symptoms of IBS-D. In the present study, we explored the therapeutic effects and the underlying mechanisms of action of the CKF formula in conjunction with PFK on IBS-D (Figure 1).

## 2. Materials and methods

### 2.1. Materials

Rabbit anti-5-HT Polyclonal (abs120892), rabbit anti-Fos Polyclonal (abs131453), rabbit anti-ZO-1 Polyclonal (abs131224) and rabbit anti-occludin Polyclonal (abs136990) antibodies were obtained from Absin Bioscience Inc. (Shanghai China). ELISA kits for rat substance P (SP) and vasoactive intestinal peptide (VIP) were purchased from the Elabscience Biotechnology Co., Ltd. (Wuhan, China). Hematoxylin-eosin dyeing solution was acquired from Wuhan Boster Biological Engineering Co., Ltd. (Wuhan, China). All other chemicals were purchased from Xilong Chemical Co., Ltd., (Guangdong China). Bifid triple viable capsule (PFK) was purchased by Shanghai Xinyi Pharmaceutical Co., Ltd. Target-colon probiotics capsule is a colon positioning release capsule, with a national invention patent (CN00117989.6).

### 2.2. Animals

Male-specific pathogen-free (SPF) Wistar rats (6–8 weeks) were supplied by SPF (Beijing) Biotechnology Co., Ltd. (Beijing, China). Rats were housed at the SPF experimental animal center, Jiangsu Academy of Traditional Chinese Medicine at 23 ± 3°C and 55 ± 5% humidity under a circadian light–dark cycle (12 h each). The animals were nourished with water and standard mouse chow *ad libitum*. All animal treatments have been supervised and approved by the Animal Ethics Committee at Jiangsu Academy of Traditional Chinese Medicine (AEWC-20220611-212).

### 2.3. Preparation of CKF and Senna

The medications adopted the famous Traditional Chinese Medicine prescription “Chang-Kang-Fang (CKF),” which has obtained the national invention patent (ZL201110336380). CKF was prepared from following listed raw material (Table 1), purchased from the Pharmacy of Jiangsu Province Hospital on Integration of Chinese and Western Medicine and accredited by Professor Songlin Li following the standards of the Chinese Pharmacopoeia (2010 edition). The seven herb samples have been deposited at Department of Translational Medicine, Jiangsu Academy of Traditional Chinese Medicine with voucher numbers: No. 20190910 for Paeoniae Alba Radix, No. 20190911 for Saposhnikoviae Radix, No. 20190912 for Rehmanniae Radix, No. 20190913 for Cuscutae Semen, No. 20190914 for Coptidis Rhizoma, No. 20190915 for Fagopyri Dibotryis Rhizoma, No. 20190916 for Periostracum Cicadae. The methods for identifying and quantifying the chemical profiles of CKF have been reported in previous studies (Mao et al., 2017). The seven crude herbs were decocted in 10 and 8 vol of distilled water (1.5 h each time), respectively. Then, the twice decoctions were combined and concentrated to 0.5 g crude drug per mL and stored in the refrigerator at 4°C for further use. Senna (1310050432) was provided by Huqiao Pharmaceutical Co., LTD, (Bozhou, China). Senna was soaked in boiled water five times and left overnight. After filtering, the filtrate was concentrated to 0.3 g/mL crude drug for intragastric administration.

### 2.4. Experimental design

**Figure 2:**
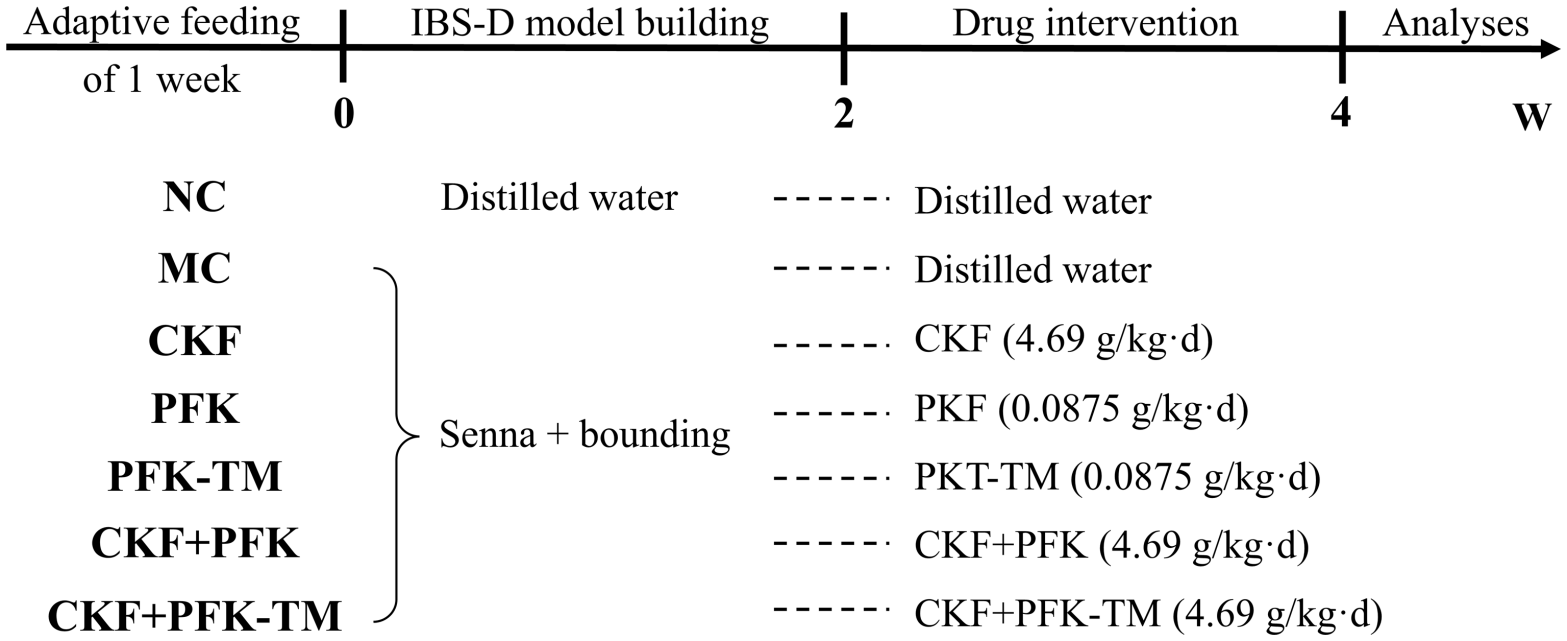
The schematic illustration of the experimental design. NC: Control group; MC: Model control group; CKF: Chang-Kang-Fang group; PFK: Bifico group; PFK-TM: Bifico target-colon capsule group; CKF+PFK: Chang-Kang-Fang combined with Bifico group; CKF+PFK-TM: Chang-Kang-Fang combined with Bifico target-colon capsule group.

After 1 week of confirmation, rats were randomly divided into seven groups (*n* = 10 per group): (1) NC group; (2) MC group; (3) CKF group; (4) PFK group; (5) PFK-TM group; (6) CKF + PFK group; (7) CKF + PFK-TM group. The modeling methods of IBS-D made a little modifications refer to the reference (Al-Chaer et al., 2000). Specifically, all groups except the NC group was orally administrated with 0.3 g/mL senna and then restraint stress for 2 weeks, and the NC group was given sterile water. The methods of restraint stress were as follows: the limbs of rats were restrained by home-made container for a duration of 2 h for 2 weeks, starting 1 h after the gavage, to produce certain stimuli. After model establishment, the NC group and MC group were intragastrically administrated sterile water. The treatment groups were gavage with 4.96 g/kg CKF, 0.0875 g/kg PFK and PFK-TM or their combination for 2 weeks at 9:00 am. The dosage of rats was calculated according to “equivalent dose method of animal body surface area ratio conversion,” and one capsule was administered each time. During the experiment period, the mental status, activity, hair, vision, diet and defecation were recorded daily and the growth rate of the weight was calculated. Weight growth rate = (weight on the day − initial weight)/initial weight × 100%. The schematic diagram of the experimental process is displayed in Figure 2.

At the terminal of the experiment, all rats were anesthetized with inhaling 2% isoflurane. Blood samples were taken from the abdominal aorta, centrifuged at 3000 rpm for 15 min after standing at room temperature for 3 h and the supernatant was collected as blood serum. Rats were euthanized by 5% isoflurane. The fresh colon and whole brain tissues were promptly excised and divided; one part was fixed in 4% paraformaldehyde for at least 24 h and then embedded in paraffin wax; another part was placed on the ice and then stored at −80°C for further analysis.

### 2.5. Assessment of the AWR score

The abdominal withdrawal reflex (AWR), is a reliable index for estimating changes in visceral hypersensitivity. Based on the procedure previously described (Choi et al., 2008), the rats was fasted for 12 h with unrestricted access to water, and then placed in a home-made container. Subsequently, an 8F-catheter was carefully inserted into the anus until a maker (1 cm distal from the end of the balloon) was positioned at the anus. After the rats was sedated, the balloon was slowly inflated to the pressure of 10, 20, 40, 60, and 80 mmHg lasting 20 s, respectively. Meanwhile, the reaction of rats under the different pressure was observed and scored. Each measurement was performed in triplicate. The AWR score criteria was shown in Table 2.

**Table 2:** AWR scoring criteria.

Abdominal withdrawal reflex	Score
The rats had no behavioral response to colorectal distention (CRD)	0
The rats had brief head movements followed by immobility	1
The rats contracted the abdominal muscle without lifting of abdomen	2
The rats hunched its abdomen off the ground	3
The rats’ body was arched and the pelvis was lifted off the ground	4

### 2.6. Immunohistochemical staining

After the rats were euthanized, the whole brain and an approximately 1 cm length of the proximal colon were removed, fixed in a formaldehyde solution, embedded in paraffin, sectioned and stained with IHC. After that, five fields of each sample were randomly selected, observed and captured under a microscope. The positive expression was brown-yellow staining particles; the negative expression was ‘no staining’. The mean optical density (MOD) of its positive expression for semiquantitative analysis was measured by using the Image-Pro Plus software and the average values were obtained for evaluating the levels of 5-HT and fos protein in the colon and hippocampus, as well as ZO-1 and occludin in the colon.

### 2.7. Enzyme-linked immunosorbent assay

The colon tissues were isolated, homogenized in cold PBS solution and then centrifugated at 12,000 × g for 10 min at 4°C. The supernatant was collected and the levels of SP and VIP in the colon tissues were detected using ELISA kits based on their protocols. A general protocol is as follows: (1) prepare reagents, samples, and standards; (2) add the prepared samples and standards & incubate at 37°C for 90 min; (3) wash 2×, add Biotinylated Antibody solution & incubate at 37°C for 60 min; (4) wash 3×, then add the Enzyme working solution & incubate at 37°C for 30 min; (5) wash 5×, then add the Color Reagent solution & incubate at 37°C; (6) add TMB termination solution; (7) use microplate reader to measure OD within 10 min; (8) calculate the content of samples being tested. The working range of the SP and VIP ELISA is 1,000 pg./mL-15.6 pg./mL and the minimum sensitivity value of them can reach 5 pg./mL.

### 2.8. High-throughput sequencing assay and 16S rRNA gene sequencing

Freshly rats faeces (0.5 g) were collected under sterile conditions, immediately transferred into sterile EP tube and stored at −80°C. The total genomic DNA of fecal sample was extracted DNA Extraction Kit following the manufacturer’s protocols. The DNA purity and concentration were measured by agarose gel electrophoresis. The V3–V4 region of the bacterial 16S-rDNA gene were amplified by PCR using primer pairs 343F (5′-TACGGRAGGCAGCAG-3′) and 798R (5′-AGGGTATCTAATCCT-3′). The PCR amplification of 16S rRNA gene was performed in triplicate. The PCR products were extracted from 2% agarose gel, purified with the AMPure XP beads again and quantify using Qubit dsDNA assay kit. Purified amplicons were sequenced on an Illumina MiSeq PE300 platform (Illumina, San Diego, United States). The raw reads were deposited into the NCBI Sequence Read Archive database (Accession Number: SRP421032). The raw sequencing reads were demultiplexed, quality-filtered by FASTQ version 0.20.0. Operational taxonomic units (OTUs) with ≥97% similarity were clustered and analyzed according to quantitative insights into microbial ecology (QIIME) package. Meanwhile, the chimeric sequences were detected and removed using UCHIME. The taxonomy of each OTU representative sequence was analyzed by RDP Classifier version 2.2 against the Silva (v123) database with a confidence threshold of 70%.

### 2.9. Statistical analysis

The experimental data were presented as means ± standard error of the mean (SEM). The data were analyzed using GraphPad Prism (version 8.0.2). Statistical analysis was conducted using one-way ANOVA. The t-test was used to compare the differences between each two groups. *p*-value < 0.05 was judged as statistically significant.

## 3. Results

### 3.1. Rat model of IBS-D

Considering the death of 3 rats during the pre-experiment stage, a total of 95 rats were used in our experiment, including 83 rats in the model group and 12 rats in the control group. During the experiments, three rats died in the model group. The death of one rat could have been due to improper posture and strength when restrained. The cause of death of the other two rats might have been unskilled gavage or the reduced diet. Eleven rats in the MC group were eliminated because there was no significant difference compared to the NC group, and the remaining 69 rats were included for the experiments. After modeling, two rats from the NC and MC groups were used to study the pathological characteristics of the colon. The success rate of establishing the IBS-D animal model in the NC and MC groups is presented in Table 3.

**Table 3:** Success rate of modeling.

	Before modeling (*n*)	Death (*n*)	Mortality rate during modeling (%)	After modeling (*n*)	Success rate of modeling (%)
NC	12	0	0	12	–
MC	83	3	3.61	69	83.13

### 3.2. Effect of the CKF formula combined with PFK on rats with IBS-D

**Figure 3:**
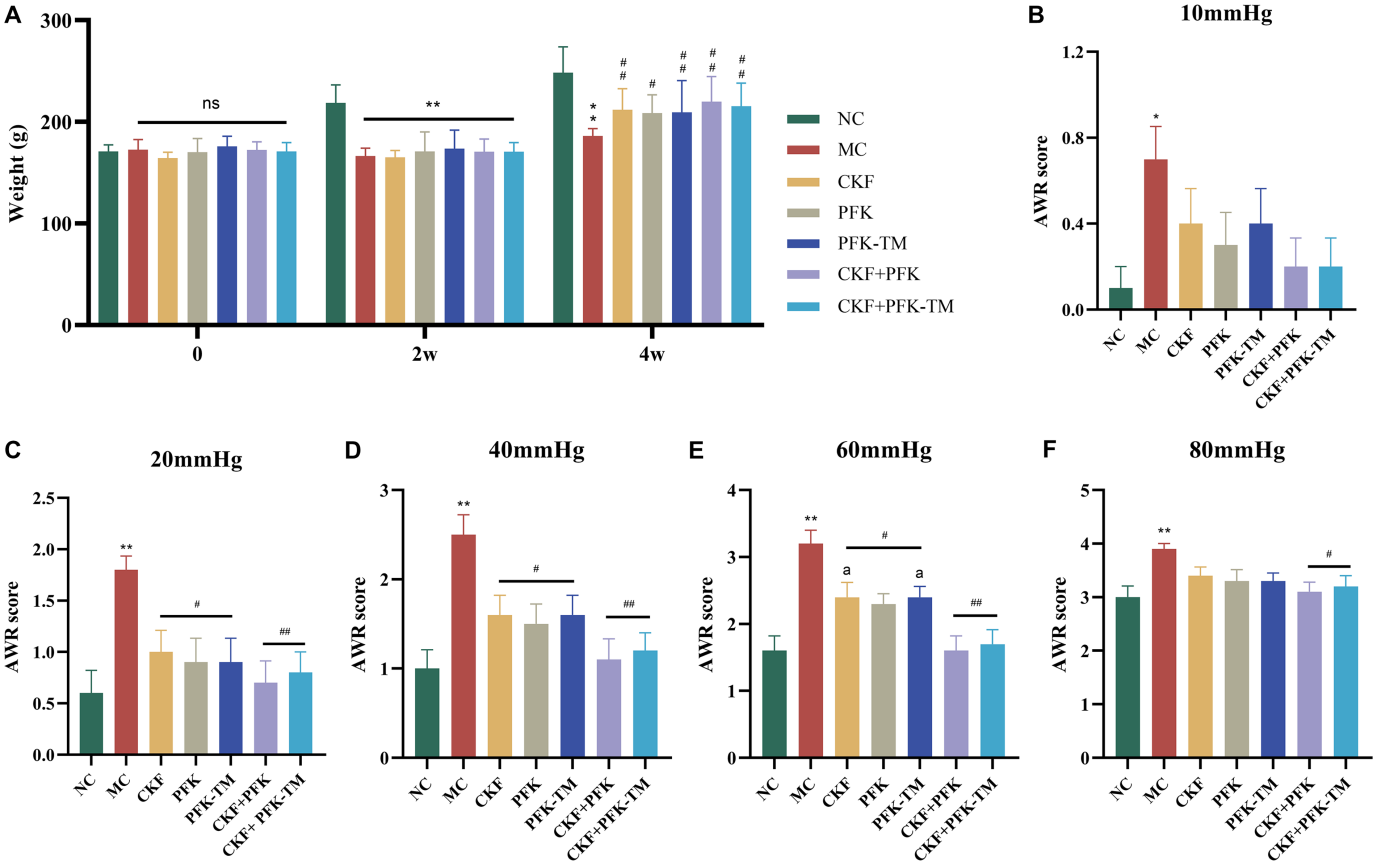
Effects of the CKF formula combined with PFK on the body weight and AWR score in IBS-D rats. **(A)** The body weight. **(B)**–**(F)** The AWR score under 10, 20, 40, 60, and 80 pressure in seven groups, respectively. The data were represented as mean ± SEM. *p < 0.05, **p < 0.01 versus NC group; ^#^*p* < 0.05, ^##^*p* < 0.01 versus MC group; ^a^*p* < 0.05 versus CKF + PFK group. NC, control group; MC, model control group; CKF, Chang-Kang-Fang group; PFK, Bifico group; PFK-TM, Bifico target-colon capsule group; CKF-D + PFK, Chang-Kang-Fang combined with Bifico group; CKF-D + PFK-TM, Chang-Kang-Fang combined with Bifico target-colon capsule group; AWR, Abdominal withdrawal reflex.

The morphological characteristics of rats, defecation and the AWR score were observed to assess the effect of the CKF formula combined with PFK on rats with IBS-D (Figure 3). The rats in the control group were in a spirited mental state, had smooth and glossy hair, moderate body size and were agile in their activities. However, the rats in the model group demonstrated a poor mental state and wizened hair. Compared with the MC group, the above features showed obvious improvement in the five intervention groups. Moreover, the body weights of the rats in the treatment groups were significantly increased compared with the MC group; however, there was no statistically significant difference (Figure 3A). Based on the Bristol score scale (Table 4) (Lewis and Heaton, 1997), the rats in the NC group had normal feces, whereas the feces of rats in the MC group were sparse and unformed. After the intervention, the symptoms of diarrhea were gradually disappeared and the form of stool reversed to normal.

**Table 4:** The Bristol stool scale.

Grade	The Bristol stool scale	Score
1	The faeces are scattered hard lumps, similar to nuts.	1
2	The faeces are sausage-shaped but lumpy.	2
3	The faeces are sausage-shaped but with cracks on its surface.	3
4	The faeces are sausage-like or snake-like, smooth and soft.	4
5	The faeces are soft mass with clear-cut edges.	5
6	The faeces are fluffy pieces with indistinct edges, a mushy stool.	6
7	The faeces are watery, no solid pieces, completely liquid.	7

Under a balloon pressure of 10, 20, 40, 60, and 80 mmHg, the AWR scores of rats in the MC group were markedly greater compared with that of rats in the NC group, indicating high visceral hypersensitivity among rats in the MC group (Figure 3). Compared to the MC group, there was no significant difference in the AWR score of rats in the treatment groups under a pressure of 10 mmHg; however, there were significant differences under pressures of 20, 40, and 60 mmHg. Unexpectedly, only the CKF + PFK and CKF + PFK-TM groups demonstrated a statistical discrepancy under a pressure of 80 mmHg. Most importantly, the combination of CKF + PFK exhibited a downtrend in the AWR score compared with the use of CKF formula or PFK-TM alone under a pressure of 60 mmHg (Figures 3B–F). These results suggested that the combination therapy of CKF + PFK significantly ameliorated the pathological symptoms induced by IBS-D.

### 3.3. Effects of the CKF formula combined with PFK on GI motility in rats with IBS-D

**Figure 4:**
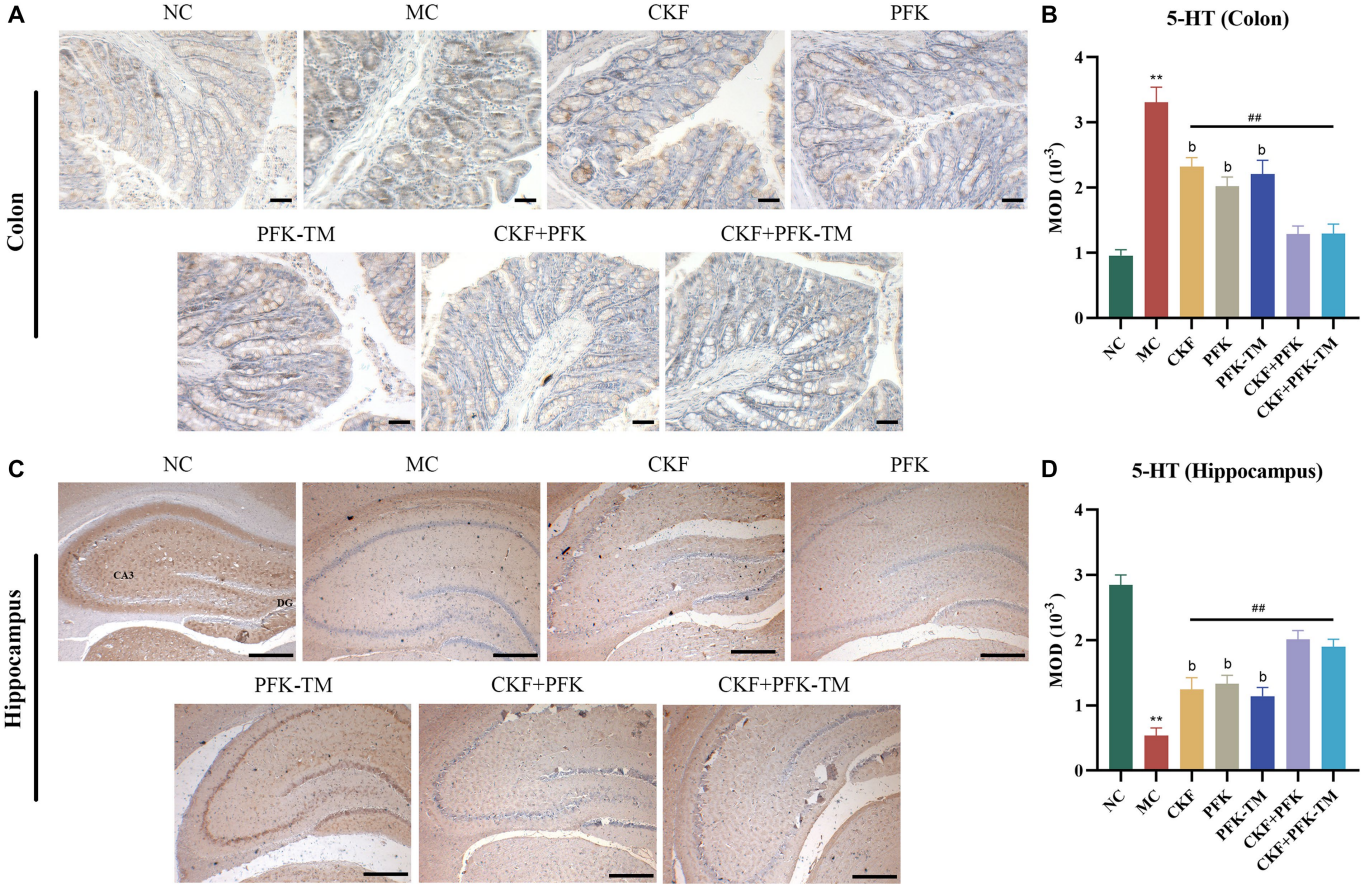
Immumohistochemical analysis stain for assessing 5-HT-positive cells expression in IBS-D rats. **(A)** IHC of 5-HT in the colon (scar bar: 100 μm), **(B)** MOD of 5-HT in the colon, **(C)** IHC of 5-HT in the hippocampus (scar bar: 1000 μm), **(D)** MOD of 5-HT in the hippocampus. The data were represented as mean ± SEM. **p < 0.01 versus NC group; ^##^*p* < 0.01 versus MC group; ^b^*p* < 0.01 versus CKF + PFK group. NC, control group; MC, model control group; CKF, Chang-Kang-Fang group; PFK, Bifico group; PFK-TM, Bifico target-colon capsule group; CKF-D + PFK, Chang-Kang-Fang combined with Bifico group; CKF-D + PFK-TM, Chang-Kang-Fang combined with Bifico target-colon capsule group; 5-HT, serotonin; MOD, mean optical density; CA3, cornu amonis 3; DG, dentate gyrus.

**Figure 5:**
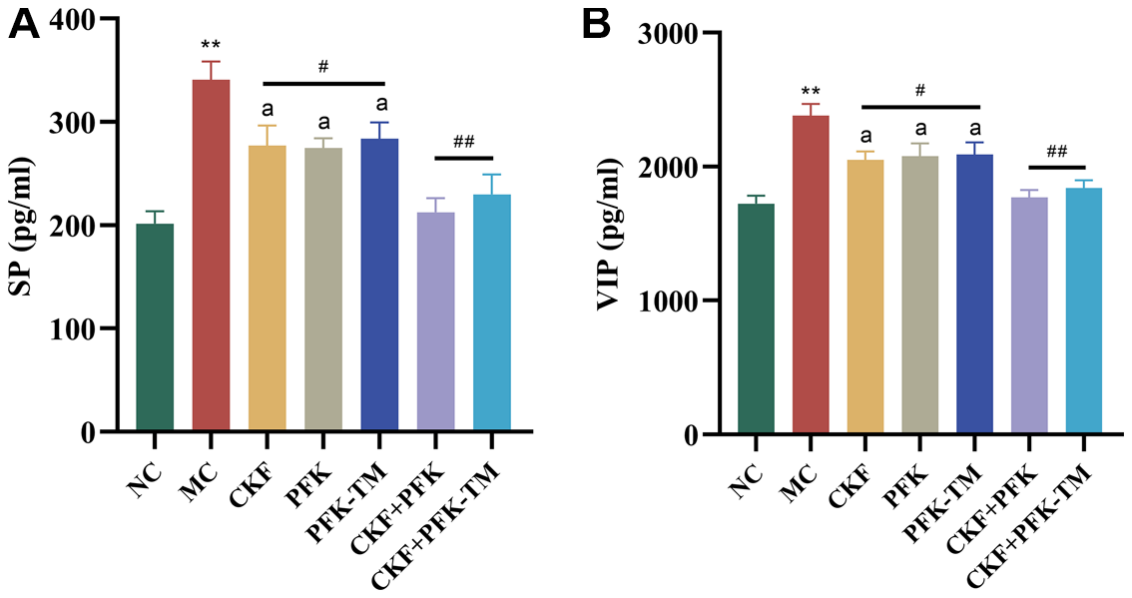
Effects of CKF + PFK on the colonic neurotransmitter levels in IBS-D rats. **(A)** The level of SP in the colon, **(B)** The level of VIP in the colon. The data were represented as mean ± SEM. **p < 0.01 versus NC group; ^#^*p* < 0.05, ^##^*p* < 0.01 versus MC group; ^a^*p* < 0.05 versus CKF + PFK group. NC, control group; MC, model control group; CKF, Chang-Kang-Fang group; PFK, Bifico group; PFK-TM, Bifico target-colon capsule group; CKF-D + PFK, Chang-Kang-Fang combined with Bifico group; CKF-D + PFK-TM, Chang-Kang-Fang combined with Bifico target-colon capsule group; SP, substance P; VIP, vasoactive intestinal peptide.

Neurotransmitters (5-HT, SP and VIP) are involved in the regulation of intestinal motility. To explore whether CKF + PFK affected GI motility in rats with IBS-D, we assessed the level of 5-HT in the colonic and hippocampal tissues as well as those of SP and VIP in the colon. The IHC results showed that 5-HT was mainly expressed in the intestinal epithelial cells and CA3 region of the hippocampus. Compared with the NC group, rats in the MC group showed strong positive staining for 5-HT in the colon and the CA3 region (Figures 4A,C) with an increasing and decreasing trend in the MOD value, respectively (Figures 4B,D). As shown in Figure 5, the ELISA revealed that the levels of SP and VIP were significantly increased among rats in the MC group compared with those in the NC group. However, these changes showed a significant reversal in all rats in the treatment groups compared with those in the MC group, especially in the combination group (*p* < 0.01), indicating that CKF + PFK is more effective than monotherapy in slowing GI motility. This finding is in accordance with that reported in the literature.

### 3.4. Effects of the CKF formula combined with PFK on visceral hypersensitivity in rats with IBS-D

**Figure 6:**
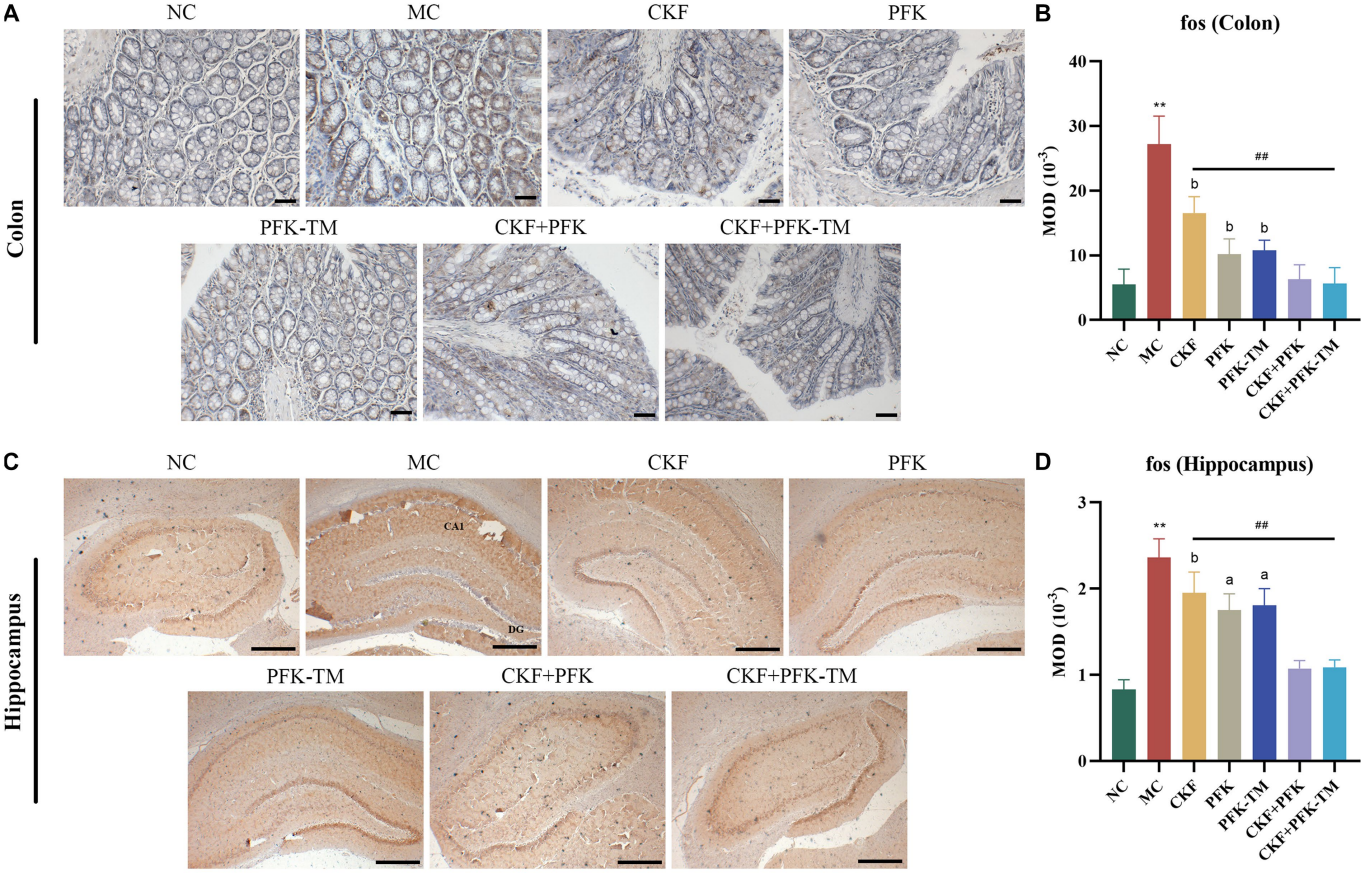
Immumohistochemical analysis stain for assessing fos-positive cells expression in IBS-D rats. **(A)** IHC of fos in the colon (scar bar: 100 μm). **(B)** MOD of fos in the colon. **(C)** IHC of fos in the hippocampus (scar bar: 1000 μm). **(D)** MOD of fos in the hippocampus. The data were represented as mean ± SEM. **p < 0.01 versus NC group; ^##^*p* < 0.01 versus MC group; ^a^*p* < 0.05, ^b^*p* < 0.01 versus CKF + PFK group. NC, control group; MC, model control group; CKF, Chang-Kang-Fang group; PFK, Bifico group; PFK-TM, Bifico target-colon capsule group; CKF-D + PFK, Chang-Kang-Fang combined with Bifico group; CKF-D + PFK-TM, Chang-Kang-Fang combined with Bifico target-colon capsule group; MOD, mean optical density; CA1, cornu amonis 1; DG, dentate gyrus.

Fos was used to explore the origin of abnormal visceral sensitivity and neural pathways associated with GI nociceptive signal transmission. Gut sensitivity has been reported to reduce significantly along with a decrease in fos mRNA to different degrees after drug interventions. The results suggested that the fos protein is mainly distributed in the cytoplasm and nuclear of the colon and the CA1 region of the hippocampus. The MC group exhibited an obvious increase in fos protein expression relative to the NC group. However, the MOD of fos was progressively declined in all treatment groups. In addition, the combination of CKF + PFK demonstrated an inhibitory effect on the expression of fos protein than monotherapy (Figures 6A–D), which indicated that CKF + PFK more strongly inhibited visceral hypersensitivity.

### 3.5. Effects of the CKF formula combined with PFK on intestinal barrier function in rats with IBS-D

Under normal conditions, the intestinal tract has an intact barrier system comprising microbial, mechanical, chemical and immune barriers. Intestinal epithelial cells and tight junctions (TJs) are the most critical components of the mechanical barrier. TJs are a specific membrane region of the apical zone of polarized epithelial cells, collectively composed of multiple transmembrane proteins and membrane-associated proteins. ZO-1 and occludin are the important component proteins of TJs (Xie et al., 2019).

**Figure 7:**
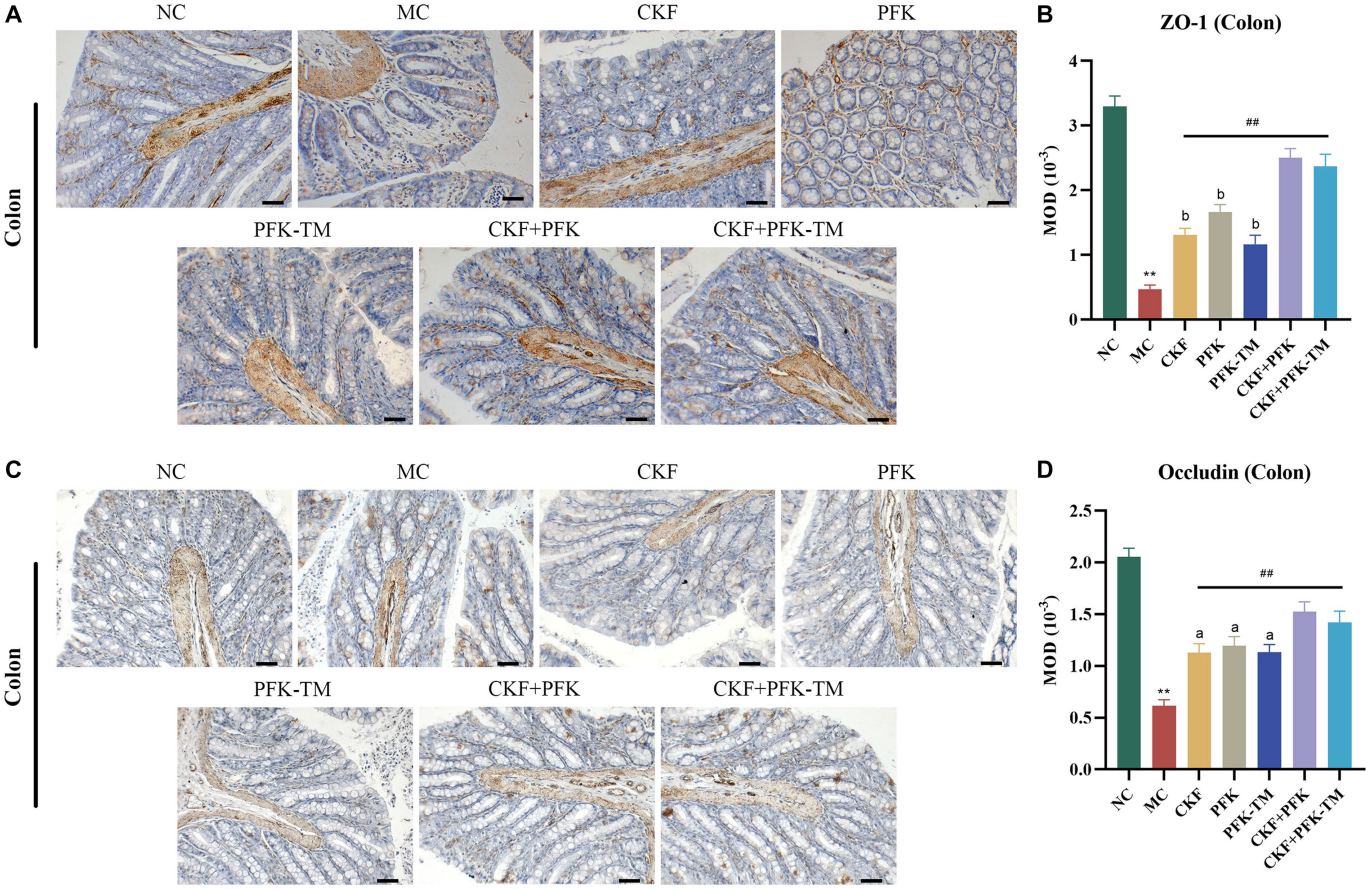
Immumohistochemical analysis stain for assessing ZO-1 and occludin-positive cells expression in IBS-D rats. **(A)** IHC of ZO-1 in the colon (scar bar: 100 μm). **(B)** MOD of ZO-1 in the colon. **(C)** IHC of occludin in the colon (scar bar: 100 μm). **(D)** MOD of occludin in the colon. The data were represented as mean ± SEM. **p < 0.01 versus NC group; ^##^*p* < 0.01 versus MC group; ^a^*p* < 0.05, ^b^*p* < 0.01 versus CKF + PFK group. All diagrams were captured at 200× magnification. NC, control group; MC, model control group; CKF, Chang-Kang-Fang group; PFK, Bifico group; PFK-TM, Bifico target-colon capsule group; CKF-D + PFK, Chang-Kang-Fang combined with Bifico group; CKF-D + PFK-TM, Chang-Kang-Fang combined with Bifico target-colon capsule group; MOD, mean optical density.

Compared with the NC group, the expressions of ZO-1 and occludin on the edge of the intestinal epithelium in the MC group were significantly decreased but markedly increased in all treatment groups (Figures 7A,B). Particularly, this effect was more evident in the CKF + PFK group than in the single drug groups, which partly repaired the TJ and protected the intestinal mechanical barrier.

### 3.6. Effects of the CKF formula combined with PFK on the composition of intestinal flora in rats with IBS-D

#### 3.6.1. The effect of bacterial diversity

**Figure 8:**
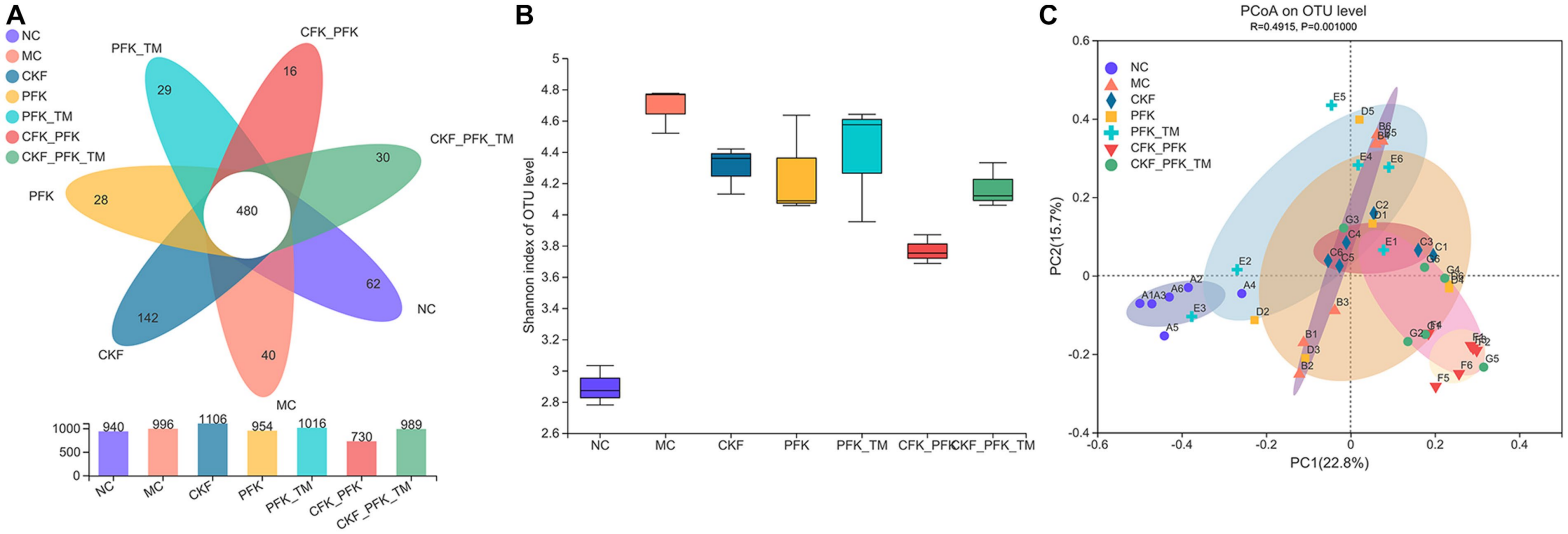
Changes in the alpha diversity and beta diversity of the gut microbiota in IBS-D rats. **(A)** Venn diagram. **(B)** Shannon (alpha diversity index). **(C)** Principal coordinate analysis (PCoA) analysis of the gut microbiota at OTU level (beta diversity index). The data were represented as mean ± SEM. NC, control group; MC, model control group; CKF, Chang-Kang-Fang group; PFK, Bifico group; PFK-TM, Bifico target-colon capsule group; CKF-D + PFK, Chang-Kang-Fang combined with Bifico group; CKF-D + PFK-TM, Chang-Kang-Fang combined with Bifico target-colon capsule group.

In our study, high-throughput sequencing of 16S rRNA was performed to evaluate the changes in intestinal microbiota after treatment with CKF + PFK. Venn diagram analysis revealed 480 common OTUs in all seven groups. A total of 940, 996, 1,106, 954, 1,016, 730, and 989 OTUs were present in the NC, MC, CKF, PFK, PFK-TM, CKF + PFK and CKF + PFK-TM groups, respectively (Figure 8A). The OTUs increased among rats in the MC group compared with those in the NC group, but decreased after treatment with CKF + PFK. The diversity of the intestinal flora was evaluated by α-diversity analysis using the Shannon index, which was increased in the MC group (*vs* NC group), as shown in Figure 8B, and decreased in the treatment groups (*vs* MC group). PCoA was performed to further study the composition of the intestinal flora in each group. The closer the points on the PCoA distribution figure, the greater the similarity of the composition of gut microbiota. The results (Figure 8C) showed that the gut microbial composition in the NC group was statistically different from that in the MC group, indicating that CKF + PFK could alter the gut microbiota induced by IBS-D.

#### 3.6.2. The changes in gut microbiota

**Figure 9:**
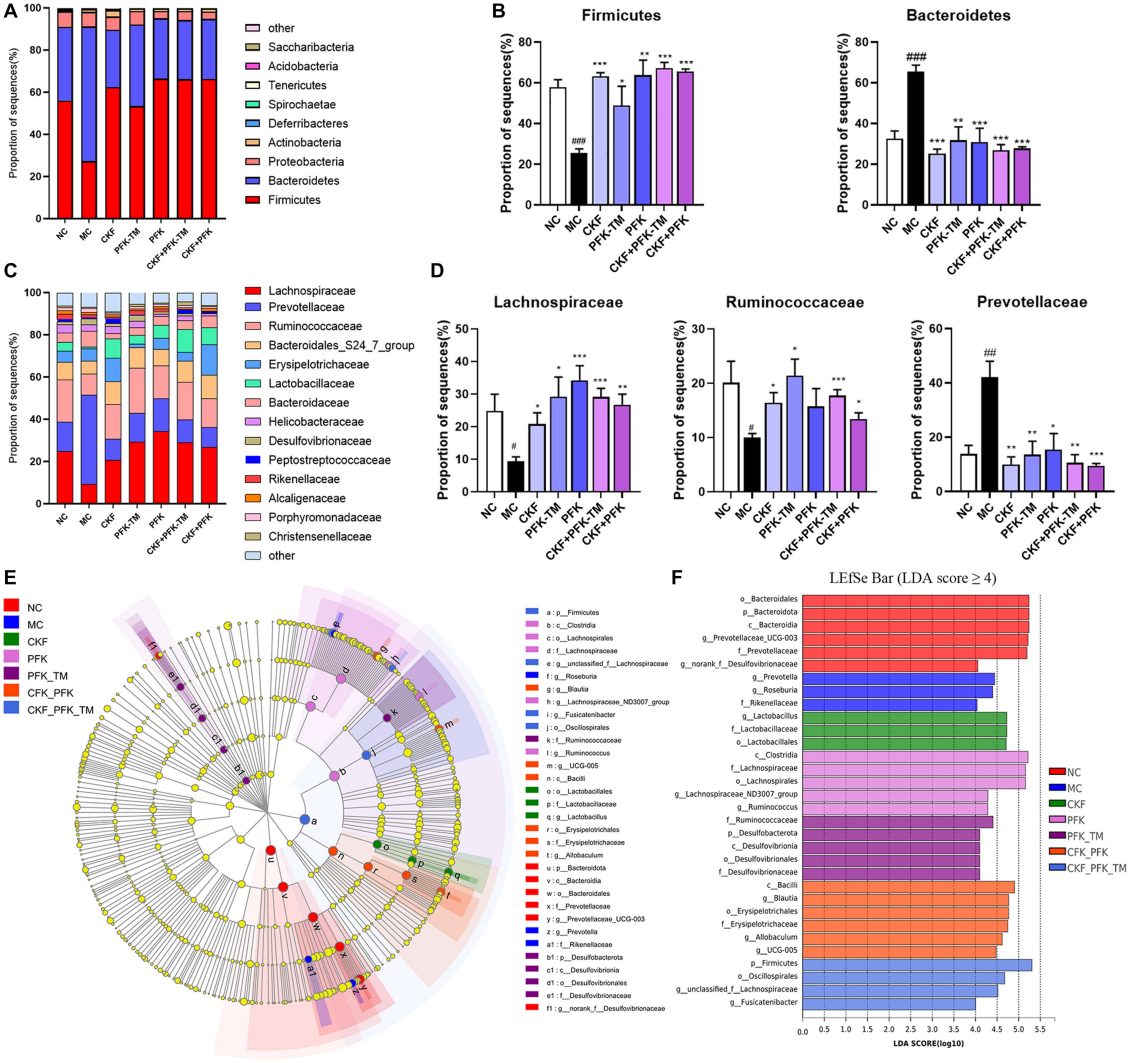
The structure of the gut microbiota and identification of specific characteristic taxa among seven groups. **(A)** Relative abundance of distinguishable phyla. **(B)** Relative abundance of Firmicutes and Bacteroidetes in seven groups. **(C)** Relative abundance of distinguishable family. **(D)** Relative abundance of Lachnospiraceae, Ruminococcaceae and Prevotellaceae in seven groups. **(E)** LEfSe tree diagram of seven groups on the genus level. **(F)** Differently abundance of intestinal bacteria taxa was obtained by linear discriminant analysis (LDA) score. The data were represented as mean ± SEM. ^#^*p* < 0.05, ^##^*p* < 0.01, ^###^*p* < 0.01 vs. NC group, *p < 0.05, **p < 0.01, ***p < 0.001 vs. MC group. NC, control group; MC, model control group; CKF, Chang-Kang-Fang group; PFK, Bifico group; PFK-TM, Bifico target-colon capsule group; CKF-D + PFK, Chang-Kang-Fang combined with Bifico group; CKF-D + PFK-TM, Chang-Kang-Fang combined with Bifico target-colon capsule group.

At the phylum level, 90% of the intestinal microbiota was composed of *Firmicutes*, *Bacteroidetes*, and *Proteobacteria* in all groups. A decrease in the abundance of *Firmicutes* and an increase in *Bacteroidetes* were observed in the MC group, which were significantly reversed by the CKF formula, PFK or their combination treatment (Figures 9A,B). At the family level, an increased abundance of *Lachnospiraceae* and *Ruminococcaceae* was observed in all treatment groups; however, the populations of *Prevotellaceae* decreased in rats with IBS-D (Figures 9C,D). The linear discriminant analysis effect size (LEfSe) method was used to identify the dominant bacterial taxa among the seven groups from the phylum to genus levels (LDA score ≥ 4). As illustrated in Figures 9E,F, the dominant types were *o_Bacteroidales*, *p_Bacteroidota*, *c_Bacteroidia* and *g_Prevotellaceae_UCG-003* in the NC group, and *g_Prevotella* and *g_Roseburia* in the MC group. However, there was an obvious discrepancy in the CKF formula, PFK and the combination treatment groups. In the CKF formula group, *g_Lactobacillus*, *f_Lactobacillaceae* and *o_Lactobacillales* played major roles. A rich abundance of *c_Clostridia*, *f_Lachnospiraceae* and *o_Lachnospirales* was observed in the PFK group. The PFK-TM group demonstrated enrichment of *f_Ruminococcaceae* and *p_Desulfobacterota*. A dramatic enrichment in *c_Bacilli*, *g_Blautia*, *o_Erysipelotrichales* and *p_Firmicutes*, as well as *o_Oscillospirales* and *f_Lachnospiraceae* was observed in the CKF + PFK and CKF + PFK-TM groups, respectively.

## 4. Discussion

The present study demonstrated that the combination of the CKF formula and PFK had superior efficacy than monotherapy in ameliorating the symptoms of diarrhea and abdominal pain in IBS-D. The potential mechanisms were associated with retarding intestinal motility, reducing visceral hypersensitivity, enhancing intestinal barrier function and shaping the composition of the GI microbiota. Previous study has showed that the components detected by CFK contained alkaloids, flavonoids, polysaccharides and etc. (Mao et al., 2017). Studies have reported that alkaloids and flavonoids could regulate the composition of intestinal microbiota (Zhang et al., 2012; Espley et al., 2014). Polysaccharides, as a prebiotic, generally have beneficial effects on the gut microbiota. Therefore, we speculate that the alkaloids, flavonoids, and polysaccharides from CKF could have synergistic effects with PFK.

It is widely recognized that the pathogenesis of IBS is complicated, and monotherapy for IBS-D has limited clinical application due to unsatisfactory efficacy. Therefore, combination therapies are often applied clinically to achieve better synergistic efficacy. Ko et al. (2011) reported that Huoxiang-zhengqi-san combined with Duolac 7S had superior synergistic curative efficacy than monotherapy in IBS-D. At present, co-treatment with CHMs and probiotics has been reported to distinctly improve various symptoms in patients with IBS-D with decreased adverse reactions (Chapman et al., 2011; Tang et al., 2018; Zhou et al., 2019; Zheng et al., 2021). Our study revealed that the effectiveness of combination therapy was better than monotherapy in ameliorating prominent symptoms of IBS-D. Thus, we propose that a combination of CHMs and probiotics may be a promising and beneficial therapeutic strategy for IBS-D.

5-HT is primarily secreted from the EC cells located in the intestinal mucosa, which plays an essential role in modulating GI motility, intestinal secretion and afferent signaling to the central nervous system (CNS) (Spiller, 2007; Zhu et al., 2017). Studies have reported that the 5-HT levels in the intestinal mucosa was elevated in patients with IBS-D (Bearcroft et al., 1998; Houghton et al., 2003; Gao et al., 2022). Our study revealed that the combination treatment of CKF + PFK resulted in significantly decreased 5-HT levels in the colon than monotherapy (Figures 4A,B), suggesting that the possible mechanism of the combined therapy in ameliorating the symptoms of IBS-D may be related to the decrease in 5-HT levels. Serotonin transporter (SERT) can rapidly re-uptake 5-HT in the effective site and regulate GI movement. Excess 5-HT contributes to abdominal pain, diarrhea and bloating, among other symptoms (Geng et al., 2020). Furthermore, a decreased expression of SERT was observed in patients with IBS-D and SERT-knockout mice exhibited high visceral hypersensitivity with watery stools, indicating that SERT may be a promising therapeutic target for IBS-D (Gao et al., 2022). Studies have been reported that Tong-Xie-Yao-Fang could decrease the level of 5-HT by elevating the SERT mRNA expression in the intestine (Houghton et al., 2009; Foley et al., 2011; Min and Rhee, 2015; Hou et al., 2018; Li et al., 2018). Interestingly, our previous research suggested that CKF could increase the expression of SERT (Fan et al., 2019). However, whether CFK + PFK is more effective at upregulating the expression of SERT and consequently reducing the 5-HT levels than monotherapy is worthy of further study.

Most IBS patients experience anxiety and depression. The occurrence of anxiety and depression could reduce the level of 5-HT in the hippocampus (Flores-Ramos et al., 2014), indicating IBS might decrease the 5-HT levels in the hippocampus. This present study showed that the combination of CKF and PFK improved the symptoms of IBS-D, thereby alleviating anxiety and depression, leading to an increase in 5-HT levels in the hippocampus (Figures 4C,D).

Visceral hypersensitivity is a clinical marker for the diagnosis of IBS-D, the pathogenesis of which is closely related to the interaction between nerve growth factor (NGF) and the enteric nerve. Generally, NGF interacts with mast cells and sensory nerve fibers to mediate visceral hypersensitivity (Xu et al., 2016), and it can rapidly induce the expression of fos mRNA and protein (Kruijer et al., 1985). Fos, an early gene, has been used for the measurement of the response to pain stimulus in various studies (Liu et al., 2011). Clinical experiments have confirmed that the elevated expression of fos mRNA in the colon could increase the severity of pain (Cibert-Goton et al., 2021). Notably, combined therapy with CKF and PFK could lower the level of fos than monotherapy (Figure 6). Therefore, whether this combination therapy reduces visceral hypersensitivity by decreasing the level of fos is worthy of further investigation.

Presently, researchers are increasingly focusing on the mechanisms by which the intestinal barrier affects IBS-D (Chao et al., 2021). Intestinal permeability is a functional characteristic of the intestinal barrier, which is regulated by TJs in epithelial cells (Linsalata et al., 2020). A study found that the downregulation of ZO-1 and occludin levels in patients with IBS-D was associated with increased colonic paracellular permeability (Hou et al., 2017). Probiotics could upregulate the expression of TJs to enhance intestinal barrier function and improve diarrhea (Nébot-Vivinus et al., 2014). The present study demonstrated that CKF + PFK therapy enhanced the levels of ZO-1 and occludin compared with monotherapy (Figure 7). Taken together, the alleviative effect of the combined therapy on IBS-D may be partially associated with augmenting intestinal barrier function.

Recently, a great deal of evidence has revealed that dysbacteriosis may promote the development of IBS, particularly IBS-D (Skrzydlo-Radomanska et al., 2021). Probiotics supplementation (e.g., *Lactobacillus*) could alter the intestinal microbial composition and ameliorate the symptoms of IBS-D (e.g.: bloating) (Compare et al., 2017). In our study, a reduced relative abundance of *Prevotellaceae* and elevated proportions of *Lachnospiraceae* and *Ruminococcaceae* were observed in the CKF + PFK group (Figure 9D). This data indicated that the partial efficacy of the combined drug therapy on IBS-D might be ascribed to its ameliorative action on the regulation of the intestinal microbiota.

Small intestinal bacterial overgrowth (SIBO) is a common type of dysbacteriosis and is usually triggered by excessive bacteria in the small intestine. The GI symptoms of diarrhea, abdominal pain and bloating in patients with SIBO are similar to those with IBS-D (Ghoshal and Srivastava, 2014; Su et al., 2018). A study reported that an enriched *Prevotellaceae* enterotype could increase the incidence of IBS-D and induce low-grade inflammation (Wu et al., 2019). Our study demonstrated that the abundance of *Prevotellaceae* was increased in rats with IBS-D; however, the levels declined after the combination therapy. Thus, it can be inferred that excessive *Prevotellaceae* might aggravate diarrhea in IBS-D by causing SIBO. SCFAs are metabolites of intestinal microbial fermentation that play a crucial role in maintaining enteric health by promoting intestinal peristalsis and water absorption. In general, *Lachnospiraceae* and *Ruminococcaceae* are the producers of butyric acid (Vacca et al., 2020), and it has been reported that patients with IBS have the decreased levels of butyric acid (Zaleski et al., 2013). Our data illustrated that the combination treatment increased the relative abundances of *Lachnospiraceae* and *Ruminococcaceae*, suggesting that complementary *Lachnospiraceae* and *Ruminococcaceae* might be beneficial for patients with IBS-D by increasing the content of butyric acid.

Accumulating data have reported that the microbiota-gut-brain axis (MGBA) is the essential pathological basis of IBS-D (Li et al., 2020; Wu et al., 2022). At present, most pharmacological drugs used for the treatment of IBS-D influence the brain-gut peptides (5-HT) and gut hormones (SP, VIP) which are vital interrelated components of the MGBA (Li et al., 2020). A disorder in gut-brain interaction has been observed in IBS-D (Sabo and Dumitrascu, 2021; Mayer et al., 2022). The present study revealed that CKF + PFK therapy significantly regulated 5-HT in the colon and brain tissues as well as SP and VIP in the colon, suggesting the modulative effect on gut-brain interaction. Moreover, CKF + PFK also enriched the abundance of *Lachnospiraceae* and *Ruminococcaceae*, and consequently led to an increase in the level of butyric acid. Considering that SCFAs are recognized as a key mediator in the pathophysiological functioning of the gut-brain axis (Sadler et al., 2020; Magzal et al., 2021), further studies are required to verify whether combined treatment with CKF and PFK would have a regulatory effect on IBS-D through the MGBA.

## 5. Conclusion

Taken together, the findings of our study confirmed that the CKF formula combined with PFK has a synergistic effect on IBS-D, and the effect of the combination are better than that of monotherapy. The possible mechanism of CKF + PFK on IBS-D is related to slowing GI motility, lowering visceral hypersensitivity, enhancing intestinal barrier function and modulating the composition of the intestinal microbiota. These results suggest that the CKF formula combined with PFK may be a potential new therapeutic drug for IBS-D.

## Data availability statement

The datasets presented in this study can be found in online repositories. The names of the repository/repositories and accession number(s) can be found at: https://www.ncbi.nlm.nih.gov/, PRJNA931650.

## Ethics statement

The animal study was reviewed and approved by the Animal Ethics Committee of Jiangsu Province Hospital on Integration of Chinese and Western Medicine.

## Author contributions

ML, JZ, XYF, and JS designed the experiments. JS performed the experiments and MQZ assisted in this work. JS, MQZ, and XYF consulted and extracted the literature. YQL, WL, and TL analyzed the data and sorted out the charts. DJZ and WL reviewed and edited the final draft. XYF performed quality control. ML and JZ supervised the study. All authors contributed to the article and approved the submitted version.

## Funding

This research was supported by Jiangsu Province Traditional Chinese Medicine Science and Technology Development Project (ZD201708) (design of the study, analysis of data, collection of data), Jiangsu Cadre Health Research Project, Jiangsu Commission of Health (BJ20026) (writing the manuscript), Nanjing Lishui District Hospital of Traditional Chinese Medicine (lzy2019yj002) (interpretation of data).

## Conflict of interest

The authors declare that the research was conducted in the absence of any commercial or financial relationships that could be construed as a potential conflict of interest.

The reviewer YL declared a shared parent affiliation with the authors MZ, WL, DZ, XF, JZ, and ML to the handling editor at the time of review.

## Publisher’s note

All claims expressed in this article are solely those of the authors and do not necessarily represent those of their affiliated organizations, or those of the publisher, the editors and the reviewers. Any product that may be evaluated in this article, or claim that may be made by its manufacturer, is not guaranteed or endorsed by the publisher.
